# Thyroid cancer risk after radiation exposure in adults—systematic review and meta-analysis

**DOI:** 10.1093/jrr/rrad073

**Published:** 2023-10-09

**Authors:** Nafiseh Beygom Mirkatouli, Seiko Hirota, Shinji Yoshinaga

**Affiliations:** Graduate School of Biomedical and Health Sciences, Hiroshima University, 1-2-3 Kasumi, Minami-Ku, Hiroshima City, Hiroshima 734-8553, Japan; Research Institute for Radiation Biology and Medicine, Hiroshima University, 1-2-3 Kasumi, Minami-Ku, Hiroshima City, Hiroshima 732-8553, Japan; Research Institute for Radiation Biology and Medicine, Hiroshima University, 1-2-3 Kasumi, Minami-Ku, Hiroshima City, Hiroshima 732-8553, Japan; Research Institute for Radiation Biology and Medicine, Hiroshima University, 1-2-3 Kasumi, Minami-Ku, Hiroshima City, Hiroshima 732-8553, Japan

**Keywords:** thyroid cancer, radiation-induced, epidemiology, adult, meta-analysis

## Abstract

Notably, the growing use of radionuclear technology, especially in diagnostic and therapeutic procedures involving radiation exposure, raises concerns about the health effects of radiation. Although epidemiological studies have provided strong evidence for elevated thyroid cancer risk after radiation exposure in childhood, the risk of thyroid cancer associated with adult exposure remains to be investigated. We conducted a systematic review and meta-analysis of relevant studies on the risk of developing thyroid cancer after radiation exposure in adulthood. The PubMed and Web of Science databases were used to select eligible articles. After screening, a total of 15 studies were identified in which estimates of the standardized incidence ratio (SIR) and the relative risk (RR) of thyroid cancer were available in 8 and 11 studies, respectively. The overall SIR estimated by the random effects model was 2.19 [95% confidence interval (CI), 1.54, 3.10]. Cochran’s *Q* test showed significant heterogeneity in the SIRs (*Q* = 178, *P* < 0.0001). The overall RR at 10 mGy was 1.0038 (95% CI, 0.9991, 1.0085), with no significant heterogeneity (*Q* = 9.30, *P* = 0.5041). The total SIR, as well as that from each study, indicated a statistically significant excess, which could be related to screening bias. Radiation-related thyroid cancer risk was elevated in a few studies; however, the overall estimate of the RR at 10 mGy was not significant. This study demonstrates no strong epidemiological evidence for the risk of thyroid cancer in radiation exposure during adulthood; however, further research is needed.

## INTRODUCTION

The thyroid gland is one of the organs most sensitive to radiation, and the risk of thyroid cancer after radiation exposure is a public health concern. It is noteworthy that, today, with the progress and increase in the applications of radio-nuclear science in the fields of industry, energy, medical diagnosis and patient treatment, the number of exposed individuals is increasing, especially in adults.

An increased risk of thyroid cancer has consistently been shown in various populations after radiation exposure during childhood [1]. Studies on Japanese survivors of the atomic bomb clearly demonstrated that the effects of age at exposure has a significant effect on thyroid cancer risk, with a higher risk observed in individuals at exposed at a younger age [2, 3]. Studies on children exposed to medical irradiation [4–6], and residents living near the Chernobyl nuclear power plant [7–9] have shown an increased risk of thyroid cancer after childhood exposure to radiation, thereby establishing that childhood radiation exposure increases the risk of thyroid cancer.

We identified important modifiers of the dose–response association, including dose–response trends that emerged after 5–10 years of exposure, increased when exposure occurred at a younger age, declined with increasing attained age and persisted for 50 years or more after exposure [4, 10]. Furthermore, in another study of a pooled analysis of nine cohorts of populations who were exposed to radiation during childhood, the analyses reaffirmed the linearity of the dose–response as the most plausible relationship for ‘as low as reasonably achievable’ assessments for pediatric low-dose radiation (external exposure) associated with thyroid cancer risk [11].

However, findings on the risk of thyroid cancer after adult exposure to radiation are generally limited. Although several studies have reported a statistically significant increase in the standardized incidence ratio (SIR) among Chernobyl emergency workers [12, 13], the results on the association between thyroid cancer risks and radiation exposure are generally inconsistent. Furthermore, the Life Span Study (LSS) of atomic bomb survivors in Hiroshima and Nagasaki showed that adult exposure did not increase the risk of thyroid cancer [3]. Thus, the risk of thyroid cancer development associated with adult exposure remains unclear. The insufficient evidence and the importance of this issue led us to conduct a systematic review and meta-analysis of studies on the SIR and relative risk (RR) of thyroid cancer in adults exposed to radiation.

## MATERIALS AND METHODS

This systematic review and meta-analysis were performed with reference to the standard Preferred Reporting Items for Systematic Reviews and Meta-Analyses guidelines as outlined in the PRISMA 2020 statement [14] and summarized findings on adult radiation exposure and thyroid cancer risk. Exposure to radiation in adulthood was defined here as that delivered at age 18 or older because most of the occupational studies included workers who were between 18 and 20 as well as those who were 20 or older. For nonoccupational studies, we restricted and used the data on subgroups who were exposed at age 20 or older where the relevant information was available. The study populations for other studies included in this analysis were predominantly adults, and we treated these studies as those on radiation exposure in adulthood.

### Search strategy and selection of studies

**Fig. 1:**
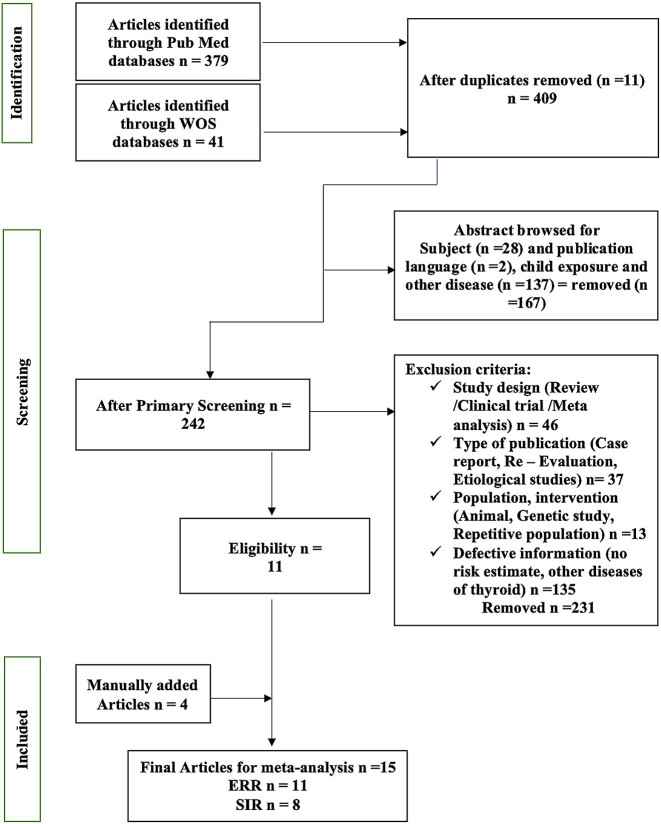
Screening and selection of studies.

Before conducting the systematic review, criteria were defined to include studies and assess their validity. We searched PubMed and Web of Science (WOS) databases, and the last search was performed on 19 April 2022. The current study used a combination of general keywords and the Medical Subject Headings (MeSH) terms to conduct a comprehensive search. We used MeSH terms of ‘neoplasms’ and ‘adult’, and general key words of ‘thyroid’, ‘radiation-induced’ and ‘epidemiology’ in PubMed searching. In searching with WOS, we used general keywords of **‘**thyroid’ (‘cancer’ or ‘neoplasm’ or ‘carcinoma’ or ‘malignancy’), ‘radiation exposure’, ‘epidemiology’ and ‘adult.’ As shown in Fig. 1, we obtained 379 articles from PubMed and 41 from WOS. Eleven articles were excluded because of duplication. A systematic review of 409 articles was initiated. In the first stage, only studies focusing on thyroid cancer in adult radiation exposure were selected for screening; in other stages, we excluded irrelevant articles that did not meet the inclusion criteria. Eligible studies were selected based on the following inclusion criteria: (i) Studies on thyroid cancer in adults exposed to radiation; (ii) Observational studies (either cohort, case–control or cross-sectional); (iii) The SIR and excess relative risk (ERR) per unit dose were available or could be calculated based on the risk and dose estimates for each dose group; (iv) Articles were published in English. Articles that corresponded to any of the following were excluded: (i) Case reports, reviews, reevaluations and etiological studies; (ii) studies without a dose assessment; (iii) in publications on overlapping populations, study updates or results of different analyses using the same dataset, only results from the most complete study were included and (iv) interventional, animal and genetic studies.

After screening using the exclusion criteria, the remaining articles were investigated and prepared for meta-analysis. Additionally, by exploring references to the UNSCEAR 2006 report on epidemiological studies of radiation and cancer [15], which provided ERR per unit dose estimates for thyroid cancer, four relevant articles were added.

### Measure of radiation effect on thyroid cancer risk

The SIR was accessible in six articles but was calculated for publications on thyroid cancer risk among medical radiation workers in South Korea [16] by dividing the observed number of cases by the expected number of cases. We first computed total SIR from sex-specific SIRs and then estimated the 95% confidence intervals (CIs) to obtain the SIR. The measure of the effect of radiation exposure on thyroid cancer risk was the ERR per unit dose to the thyroid gland, which was calculated in each study by applying a linear dose–response model. One eligible publication [17] that did not provide an ERR per unit dose estimate was calculated using a bootstrap simulation method with a linear ERR model [18]. Estimates of SIR and their 95% CIs in dose categories were used for this calculation. In another eligible publication [19], the ERR per unit dose was calculated based on the SIR estimate divided by the estimated mean dose for the study population.

### Statistical analysis

The inverse variance weighting method was applied to combine the SIR or ERR from each study using the random effects model of DerSimonian and Laird [20]. Equation (1) shows the main calculation of the overall risk estimate in the meta-analysis by studying the specific effect sizes. Equation (2) indicates the inverse of the variance of the risk estimate of study k, and Equation (3) denotes the study variance of the study effect sizes. Equation (4) defines the heterogeneity test statistic, as follows:


(1) \begin{equation*} R=\exp \left(\frac{\sum_{k=1}^n{w}_k^{\ast}\log \left({r}_k\right)}{\sum_{k=1}^n{w}_k^{\ast }}\right), \end{equation*}

(2) \begin{equation*} {w}_k^{\ast }=\frac{1}{1\!\left/ \!{W}_k\right.+{\tau}^2}, \end{equation*}

(3) \begin{equation*} {\tau}^2=\max \left[0,\frac{Q-\left(n-1\right)}{\sum_{k=1}^n{w}_k-\left({\sum}_{k=1}^n{w}_k^2\right)\!\left/ \!\left({\sum}_{k=1}^n{w}_k\right)\right.}\right], \end{equation*}

(4) \begin{equation*} Q={\sum}_{k=1}^n{w}_k{\left(\log{r}_k-\log R\right)}^2, \end{equation*}

where *R*: overall risk estimate; *r*: risk of each study, *w_k_*: inverse of the variance of risk estimate of study k, ${w}_k^{\ast }$: weight of study k [20], *n*: total number of studies k: study ID, ${\tau}^2$:between-study variance, *Q*: Cochran’s *Q* statistics.

When combining the ERR per unit dose estimates from each study, we transformed it into RR at 10 mGy and assumed that it followed a logarithmic normal distribution to obtain the standard errors of each estimate. We used an RR of 10 mGy to combine these estimates and avoid the logarithmic transformation of negative values. The mean dose of the selected articles ranged from a few tens to 200 mGy; for better coverage, 10 mGy was applied. Heterogeneity across studies was tested using Cochran’s *Q* test. All statistical analyses were performed using R version 4.2.1.

## RESULTS

### Search results

Based on their titles and abstracts, 242 relevant studies were identified by the primary screening of 409 articles, and in the intensive screening stage, exclusion criteria were applied to remove 231 articles; after manually adding 4 studies, 15 eligible studies were selected for review including 12 cohort, 3 nested case–control and no cross-sectional studies (Table 1).

**Table 1:** Characteristics of each study

First Author, Year	Country	Study population	Study design	Population size, female %	Thyroid cancer cases, female %	Age at exposure	SIR 95% CI (LL, UL)	Thyroid cancer risk 95% CI (LL, UL)	Mean thyroid dose (mGy)	Radiation exposure
Lee WJ, 2019 [16]	South Korea	Medical radiation workers	Cohort	93 922 (female % = 42.9)	827 (female % = 62.6)	NA	1.34 (1.21, 1.46) for both sexes, 1.72 (1.53, 1.91) for men, 1.18(1.08, 1.28) for women	ERR/100 mGy = 0.04 (−0.35, 0.43) for both sexes, 0.07 (−0.38, 0.53) for men −0.13 (−0.49, 0.23) for women	10.4	External/protracted
Kitahara CM, 2018 [25]	USA	Radiologic technologists	Cohort	89 897 (female % = 77.2)	476 (female % = 87.0)	NA	NA	ERR/100 mGy = −0.05 (<−0.10, 0.34) −0.06 for both sexes, (<−0.10,1.10) for men, −0.05 (<−0.10, 0.34) for women	57	External/protracted
Park S, 2020 [19]	South Korea	Radiation workers	Cohort	20 608 (female % = 13.4)	212 (female % = 10.8)	NA	1.55 (1.2, 2)	RR = 0.83 (0.49, 1.83)	14.5	External/protracted
Gudzenko N, 2022 [23]	Ukraine	Chernobyl cleanup workers	Nested case–control	149 cases and 458 controls (female % = 0)	149 (female % = 0)	18–59 years	NA	EOR/Gy = 0.40 (−0.05, 1.48)	199	External and internal /Protracted
Furukawa K, 2012 [3]	Japan	A-bomb survivors	Cohort	59 663 (female % = 64.3)	180 (female % = 88.3)	≥20 years	NA	ERR/Gy = 0.27 (<0, 0.107)	142	Acute single external
Dickman PW, 2003 [17]	Sweden	Patients with I-131	Cohort	40 535 (female % = 20.1)	104 (female % = NA)	≥20 years	1.77 (1.45, 2.14)	NA	107	Internal/protracted
Ivanov VK, 2002 [13]	Russia	Chernobyl cleanup workers	Cohort	99 024 (female % = 0)	58 (female % = 0)	NA	4.33 (3.29, 5.6)	ERR/Gy = −2.23 (−4.67, 0.22)	115	External/protracted
Kesminiene A, 2012 [12]	Belarus, Russia, Estonia, Latvia, and Lithuania	Chernobyl cleanup workers	Nested case–control	107 cases and 423 controls, (female % = 38.3)	107 (female % = 38.3)	NA	NA	ERR/100 mGy = 0.38 (0.10, 1.09)	Median = 69	External and internal/protracted
Prysyazhnyuk AY, 2018 [22]	Ukraine	Chernobyl cleanup workers	Cohort	150 813 (female % = 0)	216 (female % = 0)	NA	3.35 (2.91, 3.8)	NA	NA	External/protracted
Rahu M, 2006 [21]	Estonia and Latvia	Chernobyl cleanup workers	Cohort	10 332 (female % = 0)	14 (female % = 0)	NA	7.06 (2.8, 14.55)	NA	N.A.	External/protracted
Sigurdson AJ, 2003 [24]	USA	Radiologic technologists	Cohort	90 305 (female % = 77)	125 (female % = 86.3)	NA	1.61 (1.34, 1.88)	NA	NA	External/protracted
Boice JD, 1988 [28]	Canada, Europe, USA	Patients treated for cervical cancer	Nested case–control	150 000 (female % = 100)	NA (female % = 100)	NA	NA	RR =13.3 90% CI (0, 77)	110	External/protracted
Wang JX, 2002 [26]	China	Medical radiation workers	Cohort	27 011 (female % = 25.6)	14 (female % = 50.0)	NA	NA	O/E = 1.58 (0.9, 2.6)	244	External/protracted
Haylock RGE, 2018 [27]	UK	Radiation workers	Cohort	167 003 (female % = 9.8)	86 (female % = NA)	NA	NA	ERR /Sv =1.437 (−0.81, 8.15)	NA	External/protracted
Holm LE, 1991 [29]	Sweden	Patients	Cohort	10 646 (female % = 82)	18	NA	1.29 (0.76, 2.03)	NA	NA	Internal/protracted

NA = not available.

### Summary of each study

The eligible studies included 11 studies on radiation workers (five studies on Chernobyl cleanup workers, four on medical radiation workers and two on other workers), three on patients treated with I-131 and one on atomic bomb survivors. The dominant exposure was external (Table 1).

#### Studies on Chernobyl cleanup workers

A study of thyroid cancer incidence among cleanup workers in the Chernobyl accident was reported by Ivanov *et al*. [13]. This study included 99 024 workers living in six regions of Russia and investigated the risk of thyroid cancer from external radiation exposure. A total of 58 cases of thyroid cancer were diagnosed in the study population during 1986–1998. The mean thyroid doses were estimated to be 168 (*n* = 44 057), 93 (*n* = 35 689) and 33 (*n* = 19 278) mGy for those who first arrived at the 30-km zone in 1986, 1987, 1988 and later, respectively, which yielded a mean dose for total workers of 115 mGy. The ERR/Gy was −2.23 (95% CI: −4.67, 0.22) and demonstrated no significant relationship between thyroid cancer and radiation exposure, whereas the thyroid cancer incidence rate increased significantly in the study population compared with the general Russian population (SIR = 4.33, 95% CI: 3.29, 5.60) [13].

In another cohort study on cancer risk among Chernobyl cleanup workers in Estonia and Latvia, Rahu *et al*. [21] investigated 10 332 workers who were followed up from 1986 to 1998. The mean and median whole-body doses from the external radiation exposure were 109 and 93 mGy, respectively. The observed excesses of thyroid cancers and a significant increase in SIR (SIR = 7.06, 95% CI: 2.84, 14.55) were due to primary discernment [21].

A nested case–control study on thyroid cancer among the Chernobyl cleanup workers was conducted by Kesminiene *et al*. [12]. A total of 107 cases and 423 controls were included in the study, comprising ~66 000 workers in Belarus, 65 000 in Russia and 15 000 in Baltic countries. The overall ERR/100 mGy was 0.38 (95% CI: 0.10, 1.09), indicating a significant increase in risk with the total dose and dose from I-131, and there was a relationship between thyroid cancer and thyroid dose. Prysyazhnyuk *et al*. conducted a cohort study on thyroid cancer risk among Chernobyl cleanup workers in Ukraine [22]. In this study, 216 thyroid cancer cases were identified out of 150 813 cases between 1986 and 2012 in the base estimation of the external exposure dose. The SIR was elevated throughout the entire follow-up period, with an overall estimate of 3.35 (95% CI: 2.91, 3.80). The ERR/Gy was estimated to be 2.38 (95% CI: 0.60, 4.15) using the RADRUE method. This study provided evidence of increased rates of thyroid cancer among Chernobyl cleanup workers in Ukraine and demonstrated an elevated dose-associated thyroid cancer risk [22].

The last study in this group is a case–control study of thyroid cancer nested within an existing cohort of 150 813 male Chernobyl cleanup workers who participated through the Chernobyl State Registry of Ukraine, conducted by Gudzenko *et al*. [23]. This study included 149 patients and 458 controls (age range: 29–72 years). External and internal radiation exposures to the thyroid were assessed in both occupational and residential individuals, with a mean thyroid dose of 199 mGy (range: 0.15 mGy–9.0 Gy). The excess odds ratio per 1 Gy for the total dose was 0.40 (95% CI: −0.05, 1.48; *P* = 0.118), and it revealed no significant increased risk of overall thyroid cancer with a total thyroid dose.

#### Studies on medical radiation workers

In the medical radiation workers group, a study of cancer incidence in 90 305 US radiologic technologists from 1983 to 1998 was reported by Sigurdson *et al*. [24]. The SIR for thyroid cancers in both sexes was 1.61 (95% CI: 1.34, 1.88) and that for females and males was 1.54 (95% CI: 1.24, 1.83; *n* = 107) and 2.23 (95% CI: 1.29, 3.59; *n* = 17), respectively. The radiation dose was not estimated in this study. The observed increase in thyroid cancer cases may reflect, at least in part, earlier detection among medical workers with easy access to healthcare [24].

Kitahara *et al*. conducted an updated study on thyroid cancer risk among the same cohort of US radiologic technologists [25]. A total of 89 897 individuals were included in the population study, and 476 thyroid cancer cases were identified between 1983 and 2013. A mean cumulative occupational thyroid radiation dose of 57 mGy was obtained. Thyroid cancer risk, adjusted for attained age, sex, birth year, body mass index and pack-years smoked were not associated with cumulative occupational ionizing radiation dose to the thyroid gland (ERR/100 mGy = −0.05, 95% CI: <−0.10, 0.34). This study did not demonstrate a relationship between radiation exposure and the risk of adult thyroid cancer.

A cohort study of 27 011 medical X-ray workers in China between 1950 and 1995 by Wang *et al*. [26] demonstrated that the incidence of thyroid cancer was significantly higher in the earlier cohort (1950–1980). As a result, 14 thyroid cancers were found compared with 8.9 expected (RR = 1.6, 95% CI: 0.9, 2.6). Thyroid cancer risk was significantly elevated among those who began working with diagnostic X-rays before 1970 (RR =2.1, *P* < 0.05) when exposure was higher, possibly because thyroid cancer is related to occupational X-ray exposure [26].

A cohort study by Lee *et al*. [16] on cancer risk among medical radiation workers in South Korea between 1996 and 2015 identified 827 thyroid cancer cases among 93 922 workers. The thyroid dose was individually reconstructed based on badge dose measurements and survey data, with a mean dose of 10.4 mGy. Although SIR of thyroid cancer was significantly increased relative to national rates for both males (SIR = 1.72, 95% CI: 1.53, 1.91) and females (SIR = 1.18, 95% CI: 1.08, 1.28), there was no significant association between thyroid dose and thyroid cancer risk for males (ERR/100 mGy = 0.07, 95% CI: −0.38, 0.53) or females (ERR/100 mGy = −0.13, 95% CI: −0.49, 0.23).

#### Studies on other workers

A cohort study of 167 003 radiation workers, including both industrial and nonindustrial workers from the UK National Registry by Haylock *et al*. [27], investigated the cancer risk from low doses of external radiation exposure. This study showed that the risk of thyroid cancer was ERR/Sv =1.437, 95% CI: −0.81, 8.15, which improved the relationship between cancer risk and occupational external radiation exposure.

Park *et al*. conducted a study on Korean radiation workers in nuclear, industrial, medical and other occupations [19]. The radiation doses of workers, which had a population of 20 608 individuals, were collected through personal badge dosimeters; however, the thyroid doses were not available. The mean cumulative dose ± standard deviation between 1984 and 2017 (first quarter) was 11.8 ± 28.8 (range 0–417) mSv. Eighty-three cases of thyroid cancer were identified, the most frequently cited cancer (39.2%). A statistically significant increase in SIR was observed for thyroid cancer in men (SIR = 1.94, 95% CI: 1.54, 2.44, *n* = 72) but not in women (SIR = 0.92, 95% CI: 0.51, 1.66, *n* = 11). The adjusted RR of thyroid cancer in the exposed group (>0.1 mSv) was 0.83 (95% CI: 0.49, 1.83) which was not statistically significant.

#### Studies on patients

The study on radiation dose and secondary cancer risk in patients treated for cervical cancer by Boice *et al*. [28] was reported as a case–control study of 4188 women with secondary cancer among 150 000 patients from 19 population-based cancer registries from 14 countries and showed a nonsignificant 2-fold risk of radiogenic thyroid cancer. The mean age at diagnosis for cervical cancer was 52 years, and 31% of them were less than age 45 at the time of diagnosis. Their estimations were RR = 13.3 (90% CI: 0.77), and the average dose was 0.11 Gy [28].

In a Swedish study by Holm *et al*. [29], cancer incidence was studied in 10 552 patients (mean age, 57 years) who received therapeutic I-131 for hyperthyroidism, with an average follow-up of 15 years. Only 6% of the patients were under 40 years of age at the time of therapy. The mean amount of incorporated I-131 was 506 MBq; however, the thyroid dose was not estimated. This study showed a nonsignificant increase in thyroid cancer rates in comparison with that of the general population (SIR = 1.29; 95% CI: 0.76, 2.03, *n* = 18) [29].

In a cohort study by Dickman *et al*. [17], involving 36 792 individuals who were examined for I-131, the thyroid cancer risk was evaluated in relation to the thyroid dose from exposure to I-131 [17]. The mean age at exposure was 43 years for patients who had not received prior X-ray treatment and 52 years for those who had. Only 7% of patients who had not received prior X-ray treatment were <20 years of age at the time of exposure compared to 1% for those who had. The thyroid-absorbed dose of administered I-131 was estimated individually. An increased risk of thyroid cancer was observed only among the 1767 patients (mean thyroid dose from I-131 of 1.74 Gy) who reported previous external radiation therapy to the neck (SIR = 9.8, 95% CI: 6.3, 14.6), and among those originally doubted of having a thyroid tumor (SIR = 3.5, 95% CI: 2.7, 4.4) among the 11 015 patients (mean thyroid dose from I-131 of 1.37 Gy) without previous external radiation therapy. Among the 24 010 patients (mean thyroid dose from I-131: 0.94 Gy) without previous exposure to external radiation therapy to the neck who were referred for reasons other than suspicion of a thyroid tumor, no increased risk of thyroid cancer was observed (SIR = 0.91, 95% CI: 0.64, 1.26). Although the SIRs were significantly elevated in several subgroups, there was no clear association between the radiation dose to the thyroid and the risk of thyroid cancer.

#### Studies of atomic bomb survivors

Furukawa *et al*. reported the results of an analysis of thyroid cancer risk among the LSS cohort of Japanese atomic bomb survivors [3]. They analyzed data on the incidence of thyroid cancer among 105 401 members including 59 663 individuals who were exposed at age 20 or more from 1958 to 2005. A total of 371 patients with thyroid cancer were identified. The ERR at 1 Gy was estimated to be 1.28 (95% CI: 0.59, 2.70). When separate dose–response analyses were conducted by age at exposure (<20 vs ≥20), the risk for those exposed as children or adolescents was highly significant with a gender-averaged ERR/Gy of 1.36 (95% CI: 0.59, 2.7); however, no statistical evidence of a dose–response was present for those exposed as adults with ERR/Gy of 0.27 (95% CI: <0, 1.07), −0.18 (−0.24, 1.8) and 0.34 (−0.18, 1.3) for gender-averaged, male and female, respectively. The risk decreased with increasing age at exposure, and there was little evidence of increased thyroid cancer rates in patients exposed after 20 years of age.

### Statistical analysis results

**Fig. 2:**
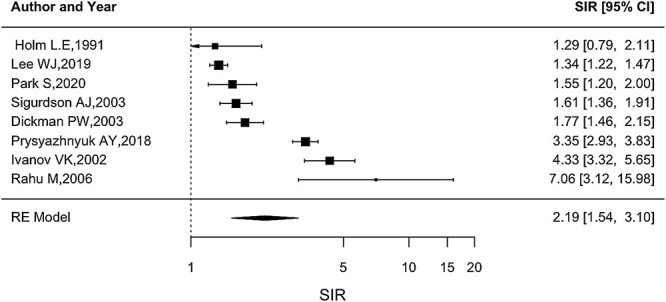
Forest plot of SIR. *SIR was calculated for publication of the Lee study [16] by using expected and observed numbers of thyroid cancer by sex. The total SIR and the 95% CIs were 1.34 (1.22, 1.47).

**Fig. 3:**
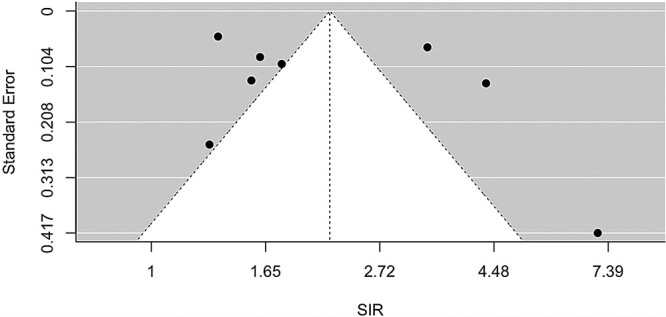
Funnel plot of SIR.

**Fig. 4:**
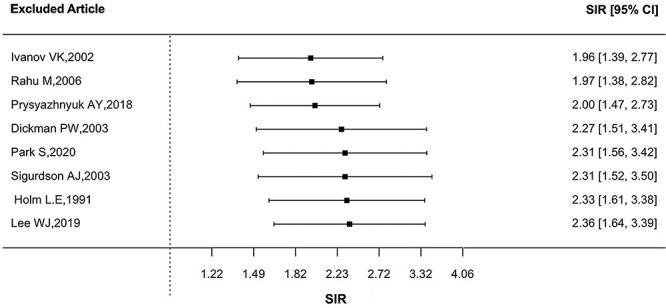
SIR sensitivity analysis.

The overall SIR was 2.19, with a 95% CI (1.54, 3.10), and Fig. 2 shows that the SIR was consistently elevated in eight studies with a significant heterogeneity (*Q* = 178, *P* < 0.0001). The funnel plot suggests that there was no publication bias (Fig. 3) with no statistical significance (p for Egger test = 0.4003). The combined SIR was higher for the three studies on Chernobyl cleanup workers (3.99, 95% CI: 2.99, 5.31) compared to the other three studies on other radiation workers (1.46, 95% CI: 1.28, 1.67) and for the two studies on patients (1.64, 95% CI: 1.26, 2.14). Sensitivity analysis showed no substantial changes in the overall SIR estimates after excluding each study (Fig. 4).

The overall estimated RR at 10 mGy was estimated to be 1.0038 [95% CI: 0.9991, 1.0085] which corresponds to an RR at 100 mGy of 1.038 (0.991, 1.085) or ERR per 100 mGy of 0.038 (−0.009, 0.085).

**Fig. 5:**
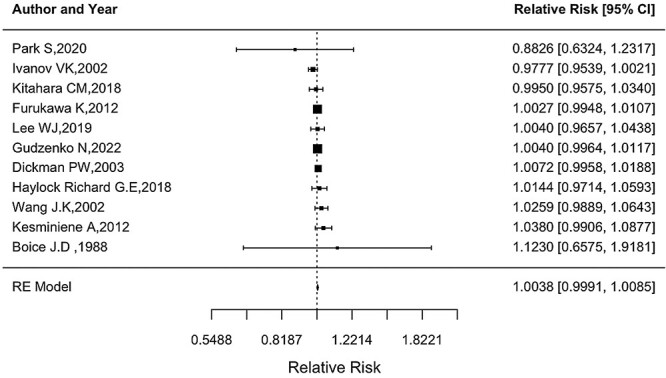
Forest plot of RR at 10 mGy *RR for Dickman *et al*. (2003) [17] was obtained according to the bootstrap simulation method using mid-doses of the thyroid dose (Gy) category (<0.25, 0.25–0.50, 0.50–1.00) and corresponding SIRs (0.98, 1.96, 1.37), which resulted in ERR/Gy of 0.7211.

**Fig. 6:**
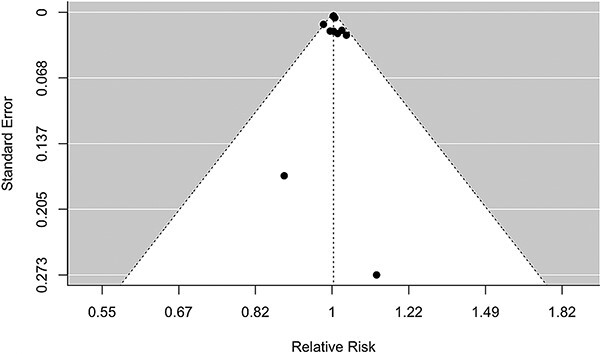
Funnel plot of RR at 10 mGy.

**Fig. 7:**
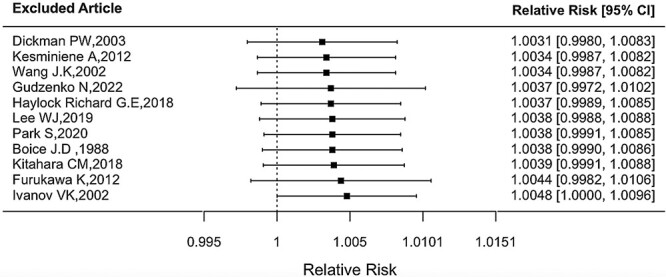
RR at 10 mGy sensitivity analysis.

As shown in Fig. 5, there was no substantial heterogeneity in the RR at 10 mGy (*Q* = 9.30, *P* = 0.5041). The funnel plot showed no publication bias (Fig. 6) with no statistical significance (*p* for Egger’s test = 0.8199). There was no substantial difference in the combined RR at 10 mGy according to the type of study population, with an estimate of 1.0009 (0.9772, 1.0252) for three studies on Chernobyl cleanup workers, 1.0095 (0.9899, 1.0294) for five studies on other radiation workers and 1.0073 (0.9958, 1.0188) for two studies on patients. Sensitivity analysis was conducted by excluding one article at a time to investigate the impact of specific studies on the overall value and did not find a notable change in the overall RR at 10 mGy, as shown in Fig. 7.

## DISCUSSION

This study, comprising 15 articles, assessed the risk of thyroid cancer after radiation exposure in adults among different populations, including Chernobyl cleanup workers, medical and other radiation workers, A-bomb survivors and patients who underwent I-131 and external beam radiotherapy, which embodies a more comprehensive assessment of thyroid cancer risk after radiation exposure. The present meta-analysis demonstrated that the RR obtained using a random effects model indicated a nonsignificant increased risk of thyroid cancer with radiation exposure at 10 mGy in adults (1.0038 [95% CI: 0.9991, 1.0085]), whereas ionizing radiation exposure has been confirmed as an established risk factor for thyroid cancer in childhood [3] with raised persistent for decades.

The overall estimate of RR at 10 mGy of 1.0038 (ERR/Gy of 0.38) was much lower than the estimate (fitted RR at 0.2 Gy of 2.7, i.e. ERR/Gy of 8.5) presented in a pooled analysis of 12 studies on thyroid cancer risk after external radiation exposure during childhood [10]. Hence, in a pooled analysis of nine cohort studies, we described the association between external radiation exposure and thyroid cancer. The evaluations proved a significant increase in RR and showed the linearity of these relationships [11]. Notably, in this analysis, we did not observe an increased risk of thyroid cancer with radiation exposure at 10 mGy. Additionally, the obtained estimation of RR in groups of studies showed no significant increase in thyroid cancer risk at 10 mGy, with no significant heterogeneity among the studies.

The pillar point that must be mentioned is the age at exposure, which is an important factor in association with thyroid cancer risk and radiation exposure; in addition to children, if individuals are exposed to radiation as adolescents, the ERR increases, and it does not decline 60 years after exposure [3]. In the current study, adult exposure was investigated, and it is notable that we explored the risk of thyroid cancer with low-dose radiation (10 mGy). In agreement with our results in studies of Chernobyl cleanup workers, there was no increased risk of overall thyroid cancer with a total thyroid dose among liquidators of the Chernobyl accident in Russia [13], although in the original information by Kesminiene *et al*. [12], a statistically significant relationship was found between thyroid cancer and thyroid dose (only in relation to internal exposure to I-131). Thyroid cancer risk increased in the dose range of over 300 mGy, which could play a role in the shape of the dose–response and the magnitude of the slope.

As discussed by Kesminiene *et al*., the magnitude of risk could be influenced by recall bias and random errors in dosimetry. In line with the results of the included studies, in a cohort study of South Korean medical radiation workers, no evidence of a statistically significant association between the thyroid dose from occupational radiation exposure and thyroid cancer rate was obtained [16]. Sensitivity analysis demonstrated nonsignificant changes, except when excluding the study by Ivanov *et al*. [13] of Russian cleanup workers, which provided the lowest estimate of RR at 10 mGy. The pooled estimate of the RR at 10 mGy was still significant.

Another key finding of our study was that the SIR consistently increased. In contrast to RR at 10 mGy estimates, there was significant heterogeneity in SIR estimates among studies. We believe that the heterogeneity in SIRs was caused by particularly higher estimates in three studies on Chernobyl cleanup workers. However, these results should be interpreted with caution because the study population is likely to have undergone more medical examinations and tests, which may have caused screening bias. A nationwide study in South Korea showed a strong correlation between the frequency of thyroid cancer screening and its incidence [30]. Thus, the increased SIRs of present study can mainly be explained by screening bias, especially since there was no significant dose–response in terms of RR at 10 mGy. Although SIR is a good measure of disease risk in comparing the study population and general population, the interpretation should be cautious because the estimates are affected by differences between the study population and general populations in various factors not only exposure of interest but also disease ascertainment methods and confounding factors.

In this study, only the SIR increased, notably in the Chernobyl cleanup workers, and the RR at 10 mGy increased in the patient group, but the increase was not significant. However, no increase in ERR/Gy was observed. Finding an association between radiation exposure and thyroid cancer in low-dose adults is difficult, and these topics are ambiguous; the association between thyroid cancer risk and low and moderate thyroid dose, the risk model, etc. Therefore, larger studies with different dose designs in various categories of adults and different applications of radiation are needed. The strengths of the current study include the following: (i) different groups of adults with radiation exposure were included and (ii) this is the first systematic review and meta-analysis of thyroid cancer in association with radiation exposure during adulthood.

The limitations of this study include the small number of eligible papers, which hampered the detailed evaluation of the effects on thyroid cancer risk according to exposure type, dose rate, dose range and origin of the study populations (race or ethnicity). Another limitation is that the adjusted confounding factors varied across the original articles, such as education level, family status, past occupational exposure to radiation, history of other cancer types or thyroid diseases, smoking and alcohol consumption.

In conclusion, the overall SIR, as well as that from each study, indicated a statistically significant excess, which could be related to screening bias. Radiation-related thyroid cancer risk was elevated in one study; however, the overall estimate of RR at 10 mGy was much lower than that from childhood exposure and was not significantly elevated. Evaluating the association between radiation exposure and thyroid cancer in adults with low-dose radiation is difficult, and larger studies with different exposure patterns in different adult groups are required.

## CONFLICT OF INTEREST

We have no conflict of interest.

## PRESENTATION AT A CONFERENCE

We have presented a part of this study in the following conferences:

- The Fourth Joint Conference of the Japanese Society of Health Physics and the Japanese Society of Radiation Safety Management.
- The Seventh International Symposium of the Network-type Joint Usage/Research Center for Radiation Disaster Medical Science, Radiation Medicine from the Perspective of Radiation Disaster Medical Science Research, poster presentation.

## CLINICAL TRIAL REGISTRATION

Not applicable.
